# Negative perfectionism and sleep quality in Chinese international students under COVID-19 epidemic: A moderated mediation

**DOI:** 10.3389/fpsyg.2022.937816

**Published:** 2022-08-01

**Authors:** Huang Zhaoyang, Chen Feng, Fan Mei, Lin Jingjing, Pan Jiyang

**Affiliations:** ^1^Department of Psychiatry, The First Affiliated Hospital of Jinan University, Guangzhou, China; ^2^School of Management, Jinan University, Guangzhou, China

**Keywords:** anxiety, COVID-19 epidemic risk perception, sleep quality, Chinese international student, moderated mediation, negative perfectionism

## Abstract

**Objective:**

This study used a moderated mediation model to test the mediating effect of anxiety on the relationship between negative perfectionism and sleep quality and the moderating effect of COVID-19 epidemic risk perception during the COVID-19 pandemic in Chinese international students.

**Materials and methods:**

A sample of 239 Chinese international students from the south of China, was surveyed with the Negative and Positive Perfectionism Scale, the Pittsburgh Sleep Quality Index, the General Anxiety Disorder Scale, and the COVID-19 Epidemic Risk Perception Inventory. Version 23.0 of SPSS and version 3.4 of PROCESS were used to perform the correlation analyses, mediation analysis, and moderated mediation analysis.

**Results:**

(1) Negative perfectionism was significantly correlated with anxiety (*r* = 0.371, *p* < 0.01) and poor sleep quality (*r* = 0.291, *p* < 0.01). Anxiety was significantly correlated with poor sleep quality (*r* = 0.594, *p* < 0.01). (2) The mediating effect test showed that anxiety had a mediating effect between negative perfectionism and poor sleep quality (β = 0.157, *p* < 0.01). (3) Epidemic risk perception moderated the mediating effect of anxiety between negative perfectionism and poor sleep quality (β = 0.070, *p* < 0.01).

**Conclusion:**

Negative perfectionism affected sleep quality indirectly through anxiety. In particular, COVID-19 epidemic risk perception moderated the relationship between anxiety and sleep quality, such that the association was stronger when the COVID-19 epidemic risk perception was high. These results provide a more comprehensive understanding of the negative link between negative perfectionism and poor sleep quality.

## Introduction

The Corona Virus Disease 2019, or “COVID-19,” has been prevalent in China and abroad for a period of time and has been affecting the development of higher education, especially international students. Following the normalization of the COVID-19 pandemic, some regions have allowed students to enter, whereas some schools internationally require students to return to complete their coursework, and many students returning from overseas or studying abroad will return to their schools, increasing their chances of getting infected as well as affecting their mental health. As a result of different events and incidents (Song et al., 2018), international students experience psychological problems, including fear, anxiety (Chen, 1999), and poor sleep quality while studying overseas (Becker and Gregory, 2020; Ingram et al., 2020; Marelli et al., 2021).

Frost defined perfectionism as a tendency to set high standards for oneself and then criticize when one fails to achieve those standards (Frost et al., 1990). Perfectionism can be divided into positive and negative forms; the former refers to individuals who set high, flexible, and realistic goals and are able to accept the consequences of failure, while the latter sets rigid, unrealistic expectations and criticizes oneself excessively when one fails to meet its goals. In a recent study, it was found that 40% of Chinese international students adjusting to American culture and dealing with the various negative emotions in a foreign country had a tendency to change from non-perfectionism to negative perfectionism and vice versa (Wang et al., 2017). When arriving in a new country, Asian international students tend to respect the collective or community culture and fulfill the social expectations that bring honor than international students with European and American cultural backgrounds (Mori, 2000; Poyrazli and Grahame, 2007). This sort of pursuit and tendency can increase international students’ academic performance and their efforts to achieve high level (Madigan, 2019), but it can create the stress (Wei et al., 2007; Nilsson et al., 2008) and anxiety of international students (Liu et al., 2021). Anxiety is an emotional state or experience that enables individuals to consciously feel a state of tension and fear and leads to feelings of helplessness and withdrawal (Bekker et al., 2003), while anxious individuals are prone to poor sleep quality (Becker and Gregory, 2020; Ingram et al., 2020; Marelli et al., 2021). Perfectionism is one of the common personality traits in the international students, especially negative perfectionists are more likely to experience anxiety (Rice and Slaney, 2002; Handley et al., 2014; Liu et al., 2021) and sleep problems during stressful events (Lundh and Broman, 2000; Akram et al., 2020), while high negative perfectionists have worse sleep quality than low negative perfectionists (Leguizamo et al., 2021). International students who have a negative perfectionism undergo heightened anxiety, which negatively impacts their sleep quality (Becker and Gregory, 2020; Ingram et al., 2020; Lai et al., 2020; Marelli et al., 2021). In fact, studies have explored the relationship between perfectionism and sleep quality. A study of perfectionism and sleep that included 346 youths found that the relationship between perfectionism and poor sleep quality was mediated by stress and poor emotional regulation (Brand et al., 2015). Similarly, a Chinese study of adolescents found that negative perfectionism and poor sleep quality were mediated by ruminative thinking (Lin et al., 2017). We deem that the effect between perfectionism and sleep quality cannot be generalized through direct effects alone. Consequently, we argue that anxiety mediates relationship between negative perfectionism and sleep quality. Therefore, we propose the hypothesis.

Hypothesis 1: Negative perfectionists in Chinese international students have worse sleep quality, with anxiety mediating the relationship.

Risk perception is the perception of an adverse situation (Horswill et al., 2004). The pandemic-related epidemic risk perception has a particularly profound impact on people during the COVID-19 outbreak, manifesting itself primarily as depression, emotional exhaustion, depersonalization, and anxiety (Grills-Taquechel et al., 2011; Yang et al., 2014; Silva-Costa et al., 2022). The more severe the catastrophic event perceived by the individual, the weaker the sense of control over the event, which in turn generates insecurity (Quan et al., 2017) and anxiety (Janssens et al., 2004). After the outbreak of the COVID-19, Chinese international students continued to experience depression and anxiety upon return (Deng et al., 2021), which led to sleep problems or made them worse (Becker and Gregory, 2020; Ingram et al., 2020; Lai et al., 2020; Marelli et al., 2021). Studies have shown that the fear and perception of COVID-19 further contributes to their anxiety (Feng et al., 2021) and affects sleep quality (Huang and Zhao, 2020) and higher levels of epidemic risk perception among international students compared to local students (Ahorsu et al., 2021). As a result, although the test of mediating effect on anxiety could explain the relationship of negative perfectionism on poor sleep quality, this mediating effect may be moderated by COVID-19 epidemic risk perception, so we propose the hypothesis.

Hypothesis 2: Anxiety among Chinese international students lead to their poor sleep quality, moderated by the epidemic risk perception.

**FIGURE 1:**
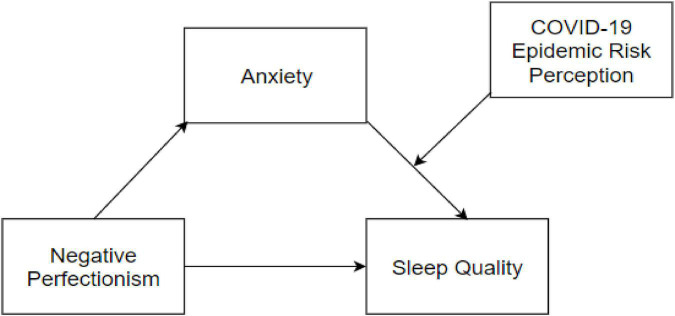
Moderated mediation model.

The study hypothesized that anxiety mediates the relationship between negative perfectionism and poor sleep quality (hypothesis 1) and that this mediating effect receives moderation by epidemic risk perception (hypothesis 2). That is, the role of negative perfectionism on sleep quality can be described by a mediating model with moderation, as shown in Figure 1. Considering that sex, first-time study abroad, study-abroad countries may be related to the variables in the model, they were included as control variables in the model test.

## Materials and methods

### Participants and procedures

The study followed a correlational design and used a web-based questionnaire as the data collection method. The questionnaires were completed through QR (quick response) codes. Participants simply had to scan the QR code, go to the on-screen questionnaire, answer the questions and click on submit. In China, QR codes are widely used as a means of accessing specified web pages or other tasks such as financial payments, enhancing identification and information searches. All participants completed the questionnaire by scanning the QR code after we explained the purpose of the study.

In this study, data related to Chinese international students who came for pre-departure medical examination between February 15 and March 26, 2022 were collected from the outpatient department of a hospital in Guangdong using questionnaire method, and a total of 260 international students were surveyed. After the data collection was completed, 21 participants were excluded due to not answering the polygraph questions correctly and taking too short (less than 130 s) or too long (more than 1,500 s) time, and the actual 239 valid questionnaires were all Chinese international students studying in Australia and the United Kingdom. The age of the participants was mainly distributed between 18 and 30 years old (217 people, 90.90%), of which 3.30% (8 people) were under 18 years old and 5.80% (14 people) were over 30 years old. There were 127 females (53.14%) and 112 males (46.86%).

### Methods

#### Positive and Negative Perfectionism Scale

The Positive and Negative Perfectionism Scale (PANPS) was developed by Terry-Short (Terry-Short et al., 1995), and the Chinese version was revised by Zhou (2012). The Chinese version consists of 25 items, which can be divided into two dimensions: positive perfectionism and negative perfectionism. Examples of PANPS scales are as follows: 1. I set high standards for myself that are almost impossible to reach (negative perfectionism). 3. I like to receive praise for my excellent performance (positive perfectionism). The PANPS scales uses a Likert-type 5-point scale, ranging from “1 = very unconforming” to “5 = very conforming.” The higher the score, the more pronounced the individual has this tendency. The internal consistency coefficients for the negative perfectionism dimension of the scale were 0.91.

#### General anxiety disorder

A self-rating scale for screening anxiety symptoms and symptom severity was developed by Spitzer et al. (2006). General anxiety disorder (GAD-7) contains a total of 7 items. Example of GAD-7 scales is as follows: 1. Feeling uneasy, worried and irritable. The GAD-7 scale is scored on a Likert-type 4-point scale. When the score is higher, the more pronounced the individual’s anxiety level is. The Chinese version of the scale was revised by Zeng et al. and has been widely used with good reliability and validity (Zeng et al., 2013), and the Cronbach’s α coefficient of this study was 0.89.

#### Pittsburgh Sleep Quality Index

Sleep quality was measured using the Pittsburgh Sleep Quality Index (PSQI). PSQI consists of 19 individual items in seven dimensions, including subjective sleep quality, sleep latency, sleep duration, habitual sleep efficiency (percentage of time in bed asleep), sleep disturbances, use of sleep medications and daytime dysfunction (each with a score of 0–3). Example of items on the PSQI scale is as follows: 18. Have you had difficulty in actively accomplishing things in the past month? The sum of the scores of the above-mentioned seven components was an overall score (the global PSQI score). Poor global sleep quality was defined by a global PSQI score of ≥ 6. The higher the score, the worse the individual’s sleep quality. The Cronbach’s α coefficient for the PSQI in this study was 0.78(Buysse et al., 1989).

#### The COVID-19 epidemic risk perception scale

The epidemic risk perception was measured using the COVID-19 risk perception scale developed by Cui et al. (2021) based on an empirical study with a large sample, which was started after the outbreak of the COVID-19, with 3 dimensions and 10 items. Example of items on the COVID-19 epidemic risk perception scale is as follows: 2. COVID-19 infection can produce serious sequelae. The higher the scale score, the stronger the epidemic risk perception. This scale was scored on a Likert-type 5-point scale. The Cronbach’s α coefficient of the scale was 0.79.

#### Data analysis

Version 23.0 of SPSS and version 3.4 of PROCESS (Hayes, 2015) were used to perform the analysis. Data for this study were collected by self-report, and therefore were tested for common methodological bias by means of the Harman single factor test before data analyzing (Podsakoff et al., 2003). Fifty-five items from the questionnaire related to the four scales were tested. The results showed that 14 factors had eigenvalues greater than 1 and these factors contributed 65.887% of the total variance. The first factor explained only 18.151% of the variance and did not reach the critical criterion of 40%, indicating that there was no significant common methodological bias in this study.

After common methodological bias evaluation, we performed descriptive statistical analysis. First, we examined the concentration and dispersion trends of the data. Then, we tested the relationships between the independent, mediating, dependent, moderating, and control variables by calculating Pearson correlation coefficients (see Table 1). After that, models were constructed based on the results of the correlation analysis, the proposed hypotheses were tested, and the mediation effect of anxiety and the moderating effect of epidemic risk perception were examined using the PROCESS (version 3.4) plug-in in SPSS.

**TABLE 1:** Correlation analysis of perfectionism, anxiety, epidemic risk perception and sleep quality.

Variables	*N*(M ± SD)	1	2	3	4	5	6	7
1. Sex	Male: 112, Female: 127	–						
2. First-time study abroad	Yes: 126, No: 113	−0.034	–					
3. Study-abroad countries	United Kingdom: 27, Australia: 212	0.062	0.047	–				
4. Anxiety	8.95 ± 2.90	−0.018	0.093	−0.248**	–			
5. Sleep quality	30 ± 7.21	−0.071	0.127*	−0.184**	0.594**	–		
6. COVID-19 Epidemic risk perception	27.11 ± 5.68	−0.065	0.103	−0.028	0.133*	0.202**	–	
7. Negative perfectionism	37.18 ± 8.88	−0.135*	0.177**	−0.082	0.371**	0.291**	0.301**	–

**At the 0.01 level (two-tailed), the correlation is significant.

*At the 0.05 level (two-tailed), the correlation is significant.

## Results

### Demographic data description

In our study, there were 126 first-time study abroad students and 113 non-first-time study abroad students in the sample of international students. The main countries where Chinese students go to study abroad include the United Kingdom and Australia, with 27 students in the United Kingdom and 212 in Australia. The age of the participants was mainly distributed between 18 and 30 years old (217 people, 90.90%), of which 3.30% (8 people) were under 18 years old and 5.80% (14 people) were over 30 years old. There were 127 females (53.14%) and 112 males (46.86%).

### Correlation analysis of perfectionism, anxiety, epidemic risk perception and sleep quality

There are significant correlations between negative perfectionism and poor sleep quality (*r* = 0.291, *p* < 0.01), anxiety (*r* = 0.371, *p* < 0.01) and epidemic risk perception (*r* = 0.301, *p* < 0.01). There’s a significant correlation between anxiety and poor sleep quality (*r* = 0.594, *p* < 0.01) and epidemic risk perception (*r* = 0.133, *p* < 0.01), a correlation between poor sleep quality and epidemic risk perception (*r* = 0.202, *p* < 0.01). Chinese international students studying abroad for the first-time showed less sleep quality problems (*r* = 0.127, *p* < 0.05). Chinese international students studying in the United Kingdom showed more anxiety (*r* = −0.248, *p* < 0.01) and sleep quality problems (*r* = −0.184, *p* < 0.01). Males had more significant negative perfectionism (*r* = −0.135, *p* < 0.01), and non-first-time international students had higher negative perfectionism (*r* = 0.177, *p* < 0.01; Table 1).

### Mediating effects of anxiety and moderating effects of epidemic risk perception

The results showed that the direct effect of negative perfectionism on sleep quality was not significant (β = 0.053, *p* > 0.05) and the inclusion of anxiety into the regression equation revealed that negative perfectionism had a significant effect on anxiety and anxiety had a significant effect on poor sleep quality, and the mediating effect was significant (β = 0.157, *p* < 0.01), demonstrating that anxiety has a mediating effect between negative perfectionism and sleep quality, with the mediating effect accounting for 74.8% of the total effect (0.210; Table 2).

**TABLE 2:** Mediating effect of anxiety.

Model pathways	Coefficient	Standardized error	*t*-value	*P*-values
Direct effect				
**Anxiety**				
Negative perfectionism	0.115	0.020	5.791	<0.01
**Sleep quality**				
Anxiety	1.375	0.144	9.546	<0.01
Negative perfectionism	0.053	0.047	1.130	>0.05
Indirect effect	0.157			<0.01
Total effect	0.210		4.097	<0.01

Control valuables: Gender, First-time study abroad, Countries to study abroad.

The product term of anxiety and epidemic risk perception was found to have a significant predictive effect on poor sleep quality (β = 0.07, *t* = 3.61, *p* < 0.001; Table 2), so epidemic risk perception had a moderating effect on anxiety and sleep quality. After a simple slope test, it was found that the effect of anxiety on sleep quality was more significant in Chinese international students with high epidemic risk perception than in international students with low epidemic risk perception, so when the level of epidemic risk perception was reduced, the effect of anxiety on sleep quality was reduced, as detailed in Table 3.

**TABLE 3:** Mediating effects of anxiety and moderating effects of epidemic risk perception.

Model pathways	Coefficient	Standardized error	*t*-value	*P*-value
**Anxiety**				
Negative perfectionism	0.115	0.020	5.791	<0.001
**Sleep quality**				
Anxiety	1.325	0.140	9.436	<0.001
Negative perfectionism	0.050	0.047	1.048	>0.05
COVID-19 epidemic risk perception	0.113	0.068	1.655	>0.05
Anxiety × COVID-19 epidemic risk perception	0.070	0.020	3.475	<0.001

Control valuables: Gender, First-time study abroad, Countries to study abroad.

## Discussion

Sleep accounts for 1/3 of a person’s life time, and sleep has an irreplaceable regulatory role in eliminating fatigue, enhancing immune function, and maintaining memory. The COVID-19 pandemic has caused heightened survival pressure among people in all countries, and people not only have to deal with illness but also with severe stress due to living in this environment for so long, leading to depression, anxiety, and poor sleep quality (Chen, 1999; Janssens et al., 2004; Grills-Taquechel et al., 2011; Yang et al., 2014; Silva-Costa et al., 2022). Globally, the World Health Organization’s 2018 survey on mental disorders among international students discovered that international student population suffers from a variety of psychological conditions as well as substance abuse caused by academic and personal pressures, which is aggravated by COVID-19 pandemic (Auerbach et al., 2018). To cope with the pressure of various aspects of international students, individuals develop a tendency to be strict with themselves, focusing on mistakes or harsh self-criticism thus increasing anxiety and thus affect sleep quality, and the hypothesis that perfectionism is closely related to sleep disorders has been widely accepted (Akram et al., 2017; Johann et al., 2017; Stricker et al., 2019). Accordingly, our study added the moderating variable of epidemic risk perception to the three, constructed a moderated mediation model, and integrated the effects of different factors (sex, study abroad factors) to examine the mediating effect of anxiety between negative perfectionism and sleep quality, and the moderating effect of epidemic risk perception. Our findings are useful for the mechanisms influencing the mental health, sleep quality, and adaptation to life abroad in the Chinese international students during the epidemic, as well as for the improvement of sleep quality and anxiety in the Chinese international students by deepening their recognition of perfectionism and regulating the epidemic risk perception.

### Perfectionist characteristics of Chinese students

Our research examined the relationship between demographic variables and perfectionism. First, sex was associated with negative perfectionism, with males exhibiting more negative perfectionism compared to females. Secondly, whether Chinese international students had studied abroad was related to negative perfectionism, meaning individuals who had studied abroad had more pronounced tendencies toward perfectionism, which was consistent with previous research (Wang et al., 2017).

### The mediating role of anxiety in negative perfectionism and sleep quality

The present study found that anxiety mediated the relationship between negative perfectionism on sleep quality by examining the mediating effect of anxiety, meaning negative perfectionism was found to affect sleep quality through anxiety, which was consistent with the results of previous studies (Akram et al., 2017, 2020), with the difference that our study removed the effect of positive perfectionism on sleep and focused only on the relationship between negative perfectionism on sleep quality. Perfectionists seem to “give up” some of their sleep quality to achieve more perfection or higher levels of performance (Akram et al., 2017; Bos and Macedo, 2018), whilst negative perfectionists seem to perform more gravely in terms of sleep (Lundh and Broman, 2000; Akram et al., 2020). Previous studies show that anxiety and sleep disorders seem to coexist and interact with each other (Becker and Gregory, 2020; Ingram et al., 2020; Marelli et al., 2021). It is also supported that perfectionism affects sleep quality, especially negative perfectionism can lead to a vicious cycle of chronic insomnia (Espie et al., 2006; van de Laar et al., 2010; Akram et al., 2020)Therefore, negative perfectionist international students are prone to apprehensive and thoughtful psychological states (Piotrowski, 2019), leading to anxiety states, which in turn affect the sleep quality. Results suggest that in order to ameliorate the anxiety and enhance sleep quality of the international students, we should start with changing their negative perfectionism.

### Moderating effect of epidemic risk perception on anxiety-mediated pathways

Studies have shown that the risk of viral infection has been affecting people’s physical and mental health after the global catastrophic event of the COVID-19 epidemic (Silva-Costa et al., 2022). Therefore, researchers conducted a series of studies in order to investigate the impact of the epidemic risk perception on physical and mental health, and found that the factor of epidemic risk perception has a significant impact on people’s lives (Yang et al., 2014; Silva-Costa et al., 2022). Due to the current pandemic, only some countries have a relatively stable epidemic situation. This environmental characteristic makes people’s perception of epidemic risk low. The perceived risk of an epidemic that occurs when international students travel from a relatively stable region to a relatively unstable country has a unique impact on mental health.

There are few studies on the epidemic risk perception among Chinese international students in the past, and the studies have been conducted in a single form, mainly on the effects of cultural adaptation or stress perception on physical and mental health of Chinese international students (Auerbach et al., 2018; Song et al., 2018) but due to the impact of the COVID-19 epidemic on the international students. In our study, the moderating effect of epidemic risk perception on anxiety and sleep quality were innovatively investigated, and the moderating effect of epidemic risk perception on the mediating process of “Negative perfectionism→Anxiety→Sleep quality” was found, specifically between the mediating variable and the dependent variable. When the epidemic risk perception was relatively high, the effect of anxiety on sleep quality was higher among Chinese international students. The effect of anxiety on sleep quality is weakened when the epidemic risk perception is low and the effect of anxiety on sleep quality is enhanced when the epidemic risk perception is high. This suggests that the process of international students having negative perfectionism to cope with high-pressure life abroad while developing anxiety that leads to insomnia is influenced by international students’ epidemic risk perception of COVID-19 that they may suffer from in the country they are going to. This relationship between anxiety and sleep quality is influenced by the increased epidemic risk perception among international students. It suggests that teaching international students who are going abroad about the COVID-19, along with teaching related precautions and hygiene habits, can help to reduce their epidemic risk perception (Leppin and Aro, 2009), which in turn can reduce the effect of anxiety on sleep quality, thus promoting the development of higher education for international students and the resolution of their mental health problems. In addition, the mediated part of the findings explored the relationship between personality traits and sleep quality, and the negative perfectionist personality was universal and therefore applicable to other groups. The moderated mediation model of our study then applies to other international students during the epidemic, those who have been living in a severe epidemic area for a long time and those who will be living in a severe epidemic area. In addition, if a global crisis like COVID-19 occurs again, this study can provide a reference and help for international student management.

### Significance and limitations

In summary, we examined the mediating effect of anxiety in the relationship between negative perfectionism and sleep quality and the moderating effect of epidemic risk perceptions on this mediating process by constructing a moderated mediation model based on the psychological characteristics and epidemic risk perceptions of Chinese international students. The innovations of this study are: 1. Perfectionism was found to be one of the common personality traits in the international students. 2. A mediation model was constructed to explore the relationship between negative perfectionism and sleep quality, and anxiety were found to mediate the relationship. 3. For the first time, epidemic risk perception was included in the study of the relationship between negative perfectionism and sleep quality among Chinese international students, and it was found that when international students had high epidemic risk perception, anxiety had a more pronounced effect on sleep quality, and vice versa. Our findings reveal the mechanism of how the epidemic risk perception affects the mental health of a group of international students, which has practical implications for improving the mental health of a group of Chinese international students. This has practical implications for improving the psychological health of Chinese international students and how to develop higher education for international students in the COVID-19 pandemic environment, which suggests that paying attention to the negative perfectionist tendencies of international students and epidemic risk perception in the current environment is beneficial for Chinese international students to adapt to life abroad, and students can be greatly motivated to return to China after graduation.

First, there are limitations in our study, as a longitudinal study was not adapted to explore the perfectionist personality traits of international students due to their mobility, and therefore deeper conclusions cannot be drawn. Second, our study only focuses on Chinese international students in some regions in the South, and does not include multiple regions for comparison at the same time. Therefore, it is hoped that future studies will be able to compare Chinese international students from different regions and provide assistance in the differentiated management of Chinese international students. Thirdly, since the Chinese international students who underwent health checkups in the outpatient clinic of the hospital in 2022 only included Chinese international students who went to two countries, including the United Kingdom and Australia. Our study’s sample lacks diversity in terms of countries of study and future study should include Chinese international students who went to different countries can be included in future studies for comparison, so as to provide targeted suggestions for managing and helping the psychological changes of Chinese international students in different regions.

## Conclusion

Our study tested a moderated mediation model to examine the relationship between negative perfectionism and sleep quality, as well as the mediating role of anxiety in both and the moderating role of perceived epidemic risk during the COVID-19 pandemic. Our findings showed that negative perfectionism predicted poor sleep quality (the more pronounced the negative perfectionism, the worse the sleep quality) and that anxiety mediated between the two. Furthermore, in the second half of the mediated pathway between negative perfectionism and sleep quality, epidemic risk perception moderated anxiety. More specifically, the predictive effect of anxiety on sleep quality was more pronounced at high levels of perceived epidemic risk.

## Data availability statement

The raw data supporting the conclusions of this article will be made available by the authors, without undue reservation.

## Ethics statement

The studies involving human participants were reviewed and approved by the Medical Ethics Committee of the First Affiliated Hospital of Jinan University. Written informed consent to participate in this study was provided by the participants.

## Author contributions

HZ, FM, and CF designed the research and reviewed and edited the manuscript. HZ carried out the literature search and data analysis. HZ, FM, CF, LJ, and PJ wrote the manuscript. All authors have read and agreed to the published version of the manuscript.
